# Economic burden of community-acquired pneumonia among elderly patients: a Japanese perspective

**DOI:** 10.1186/s41479-017-0042-1

**Published:** 2017-12-05

**Authors:** Keiko Konomura, Hideaki Nagai, Manabu Akazawa

**Affiliations:** 1 Public Health and Epidemiology, Meiji Pharmaceutical University, 2-522-1, Noshio, Kiyose, Tokyo, 204-8588 Japan; 2 Center for Pulmonary Diseases, National Hospital Organization Tokyo National Hospital, 3-1-1 Takeoka, Kiyose-shi, Tokyo, 204-8585 Japan

**Keywords:** Community-acquired pneumonia, Economic burden of disease, Cost analysis, A-DROP system, Invasive pneumococcal disease, Pneumonia

## Abstract

**Background:**

This study aimed to estimate the economic burden of community-acquired pneumonia (CAP) among elderly patients in Japan. In addition, the study evaluated the relationship between total treatment cost and CAP risk factors.

**Methods:**

An administrative database was searched for elderly patients (≥ 65 years old) who had pneumonia (ICD-10 code: J12–J18) and an antibiotic prescription between 1 June 2014 and 31 May 2015. The all-cause total healthcare costs of outpatient and inpatient CAP episodes were calculated.

**Results:**

This study evaluated data from 29,619 patients with CAP who experienced 14,450 outpatient CAP episodes and/or 20,314 inpatient CAP episodes. The mean ages were 77.5 ± 8.0 years and 81.5 ± 8.2 years among the outpatient and inpatient groups, respectively. The median treatment costs were US$346 (interquartile range: $195–551) per outpatient episode and US$4851 (interquartile range: $3313–7669) per inpatient episode. More severe cases had increased treatment costs at the treating hospitals. Male sex, diabetes, chronic obstructive pulmonary disease, and liver dysfunction were associated with increased total treatment costs, while dementia, dialysis, and rheumatism were associated with high costs of treating a CAP episode.

**Conclusions:**

The economic burden of CAP might be decreased by reducing the number of hospitalizations for mild CAP and the incidence of severe CAP. Therefore, preventative care (e.g. oral hygiene or pneumococcus vaccination) is recommended for patients with related risk factors, such as male sex, older age, diabetes, chronic obstructive pulmonary disease, liver dysfunction, rheumatism, dementia, or dialysis.

**Electronic supplementary material:**

The online version of this article (10.1186/s41479-017-0042-1) contains supplementary material, which is available to authorized users.

## Background

Community-acquired pneumonia (CAP) is a common acute infectious disease among elderly people, and is associated with high rates of hospital admission and mortality. In Japan, there are approximately 1.9 million new CAP cases every year, with approximately 70% of cases involving patients who are >65 years old and approximately 70% of these elderly patients being hospitalized [1]. Thus, pneumonia is the third leading cause of death among elderly people in Japan. Age and male sex are known risk factors for CAP among adults [2–5], and many comorbidities are also risk factors for CAP, such as chronic obstructive pulmonary disease (COPD), diabetes, cancer, dementia, congestive heart failure, and liver function failure [2, 5–10]. Prescriptions for inhaled medication or oral corticosteroids are associated with an increased risk of CAP [6]. Several methods are used to classify the severity of CAP, and the most commonly used methods for selecting the CAP treatment location are the CURB-65 and pneumonia severity index (PSI) systems. Cases with higher PSI classification (most mild: class I, most severe: class V) have increased values for mortality rate, length of stay, rate of subsequent hospitalization, and rate of admission to the intensive care unit (ICU) [11].

CAP is associated with both clinical and economic burdens, based on its high incidence, admission rate, and mortality rate [12, 13]. Many studies from various countries have estimated the economic burden of CAP among elderly individuals [14–20], and those studies have revealed that increased treatment costs are associated with older age and treatment setting [21]. For example, Dutch patients who were ≥50 years old had treatment costs of > US$5000 per CAP episode, and a Spanish population-based study revealed that direct costs for outpatient and inpatient CAP treatment were approximately US$200 and US$1700, respectively [22]. The total CAP treatment costs among inpatients increase for PSI classes I–III and reach a plateau for classes IV–V [23]. Sato et al. have also stratified the CAP-related risk based on the patient’s immune status and chronic comorbidities [12], and reported that the all-cause total healthcare costs were higher for high-risk cases, compared to low-risk cases. Although Japan is the most aged country in the world, the economic burden of CAP according to risk factors and severity remains unclear. Furthermore, it is difficult to directly compare the treatment costs in various countries because of differences in the treatment approach and insurance systems. Nevertheless, it would be useful for healthcare providers and policy makers to understand the effects of risk factors and severity on treatment costs, which could help facilitate the appropriate distribution of medical resources.

There are several prophylactic treatments for CAP. Pneumococcal disease among adults can be prevented using the 13-valent pneumococcal conjugate vaccine or the 23-valent pneumococcal polysaccharide vaccine (PPV23). Many countries, including Japan, are also introducing age-based pneumococcal vaccination programs, and the PPV23 vaccine is thought to prevent 50–85% of invasive pneumococcal disease (IPD) cases [24], although the vaccination coverage rate remains low [25, 26]. Oral hygiene is also known to reduce pneumonia onset among elderly patients [27]. Thus, based on the aging global population, a strategy is needed to reduce the economic burden of CAP by identifying patients with high predicted healthcare costs and targeting individuals who should receive prophylactic treatment. However, the effects of CAP severity and risk factors on treatment costs remain unclear, and these factors are an important part of cost-effective or cost-utility analyses. The present study used a large administrative database to estimate the economic burden of CAP among elderly Japanese patients, as well as the per-episode and total treatment costs according to disease severity and risk factors.

## Methods

### Data source

The retrospective protocol of this study was approved by the ethical committee of Meiji Pharmaceutical University (Tokyo, Japan). Patient and treatment records from 1 April 2010 to 31 May 2015 were obtained from an administrative database that is maintained by Medical Data Vision Co. Ltd. (Tokyo, Japan). Pneumonia episodes were identified between 1 June 2014 and 31 May 2015, while patient characteristics were searched up until 1 June 2014. The database includes claims data from approximately 5 million patients who received treatment at 200 acute care hospitals, and were tracked using the Japanese Diagnosis Procedure Combination/Per-Diem Payment System (DPC/PDPS). These mainly consisted of small- and medium-sized hospitals that were distributed throughout Japan. Previous reports have provided detailed explanations of the DPC/PDPS [28–31], which is a case-mix system that tracks patients based on their diagnoses and procedures. Fixed medical payments are determined according to the case-mix system and additional fees for service. In addition to the medical costs that are calculated using the DPC/PDPS, the database also contains information regarding the total healthcare costs based on the provided medical services and their related fees. Anonymized data in the database include demographic characteristics, prescriptions, diagnosis (based on the International Classification of Diseases, 10th revision [ICD-10] code), medical treatments, treatment dates, hospital admission and discharging dates, discharge status, and pneumonia severity scores at the admission based on the A-DROP system, which is advocated by the Japanese Respiratory Society [32, 33]. The A-DROP system uses a modification of the CURB-65 score, which has been adjusted to reflect Japan’s aging population. The system evaluates five factors: age of >69 years for male patients and >74 years for female patients, blood urea nitrogen of >20 mg/dL or dehydration, an SpO_2_ value of ≤90% or a PaO_2_ value of ≤60 Torr, presence of disorientation, and a systolic blood pressure of ≤90 mmHg. Cases are defined as mild, moderate, severe, or very severe, based on total scores of 0, 1–2, 3, and 4–5, respectively. The database also includes outpatient treatment records that are not covered by the DPC/PDPS.

### Definition of CAP episodes

The present study included patients who were ≥65 years old with a confirmed diagnosis of pneumonia (ICD-10 code: J12–J18) and a prescription for antibiotic treatment between 1 June 2014 and 31 May 2015. The CAP episodes were classified as outpatient episodes or inpatient episodes. The outpatient index date was defined as the date of a pneumonia diagnosis with any antibiotic prescription. The treatment period extended from the index date until the end of the antibiotic prescription. If a second antibiotic prescription was provided within 7 days after the previous prescription’s end date, the second treatment was considered part of the same outpatient episode. The inpatient index date was defined as the admission date for cases with a diagnosis of pneumonia, and the treatment period was defined as the length of stay (LOS). Inpatient CAP episodes were categorized based on treatment in the general ward or ICU (a minimum 1-night stay in the ICU). Death records (all-cause deaths based on discharge records) were only available for inpatient episodes. To ensure the study only considered cases of CAP, patients with a discharge date that was ≤14 days before the index date were excluded.

### Treatment costs

CAP-related costs can be calculated as the CAP-related healthcare costs and/or the all-cause total healthcare costs. The CAP-related healthcare costs are calculated based on treatments that are directly related to the CAP, while the all-cause total healthcare costs are calculated based on the total costs during a CAP treatment period. The former method is difficult to use for elderly patients with CAP, as they typically have one or more comorbidities, which makes it difficult to differentiate between the treatments for the CAP and the exacerbation of comorbidities. Thus, the present study used the all-cause total healthcare costs in the analyses.

All-cause total healthcare costs were calculated by combining all recorded treatment costs during a single CAP episode. The treatment costs were classified as being related to inpatient stays, office visits, drug treatments, examinations, medical procedures, and other costs. The costs for inpatient stays and office visits included the facility’s fixed costs and any service fees. The costs for drug treatments, examinations, and other costs were variable. The costs for medical procedures and other categories included service fees. A detailed breakdown of these categories is shown in Additional file 1: Table S1. Treatment costs were estimated based on medical fees and drug prices from April 2014. Because the fees and prices are revised every 2 years in Japan, the corresponding values for the period during which the treatment costs were calculated were selected.

### CAP severity

The A-DROP system scores range from 0 to 5, with the most severe cases assigned a score of 5. CAP severity was also defined based on IPD episodes (severe CAP), as the related care is typically provided in a hospital. The IPD diagnoses were classified as bacteremia or meningitis. Bacteremia cases were identified based on a bacteremia diagnosis (ICD-10: A403, A409, A419, A491, A499), blood culture records, and antibiotic drug treatment during the CAP treatment period. Meningitis cases were identified based on a meningitis diagnosis (ICD-10: G001, G009), lumbar puncture, and antibiotic drug treatment during the CAP treatment period.

### Other variables

Comorbidities were defined based on the patients’ previous medications and/or diagnoses before the index date. The present study considered diabetes (ICD-10: E11–E14), COPD (ICD-10: J42–J44), dementia (ICD-10: F00), liver function failure (ICD-10: K70–K76), rheumatism (ICD-10: M059, M060, M068, M069), and cancer (ICD-10: C00–C99, D00–D09). Liver function failure does not have any specific medications, and was defined for the study as the presence of ≥2 diagnoses before the index date. Severe renal dysfunction was identified based on at least one dialysis treatment before the index date. The detailed definitions are shown in Additional file 1: Table S2. Medications that affected pneumonia were defined as oral steroids, inhaled steroids, angiotensin-converting enzyme inhibitors, and statins. To be considered as influencing the development of CAP, these drugs had to have been prescribed at least once during the 6 months before the index date. The definitions of medications are listed in Additional file 1: Table S3.

### Analysis

All-cause total healthcare costs were calculated for outpatient and inpatient CAP episodes, as well as breakdowns of the total treatment costs. The costs of inpatient CAP episodes were also subdivided into cases that received treatment in the general ward or in the ICU. Treatment costs were also calculated according to mortality and CAP severity (based on the A-DROP score and episodes with or without IPD). The costs were stratified according to the presence of comorbidities and prescriptions, and all costs were expressed as median and interquartile range (IQR) values in US dollars (in May 2017, one US dollar equaled approximately 111.3 yen). All results were reported as mean, standard deviation, median, and IQR, as appropriate. All analyses were performed using SAS software (version 9.3; SAS Institute Inc., Cary, North Carolina, USA).

## Results

### Patient characteristics

**Fig. 1:**
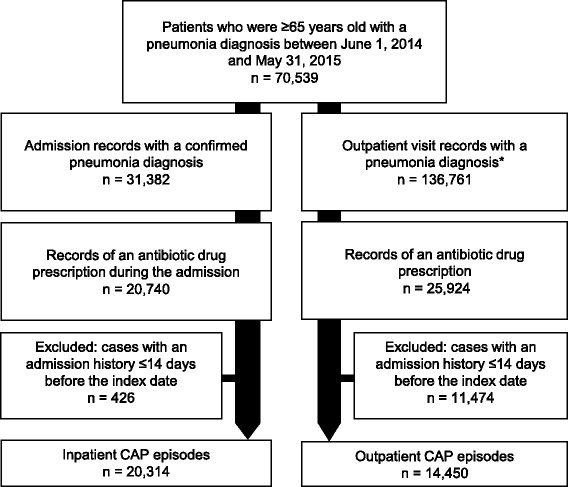
The study selection criteria. ^*^These records included both the first records of pneumonia diagnosis and subsequent follow-up visit records

There were 70,539 patients who were ≥65 years old and had a pneumonia diagnosis in the database. The study evaluated data from 29,619 patients with CAP, who experienced 14,450 outpatient CAP episodes and/or 20,314 inpatient CAP episodes. The study selection criteria is shown in Fig. 1. The mean ages were 77.5 ± 8.0 years and 81.5 ± 8.2 years among the outpatient and inpatient groups, respectively. Across both treatment settings, 61% of the patients were men (Table 1). The median CAP treatment periods were 7 days (IQR: 4–9 days) per outpatient episode and 14 days (IQR: 9–25 days) per inpatient episode. Liver function failure was the most prevalent comorbidity among both inpatient and outpatient cases, and was followed by COPD. Outpatient episodes were associated with a higher rate of cancer, compared to inpatient episodes (16% vs. 7%).

**Table 1:** Patient characteristics

	All patients		Outpatient CAP episodes		Inpatient CAP episodes	
	*n* = 29,619		*n* = 14,450		*n* = 20,314	
Age in years, mean (SD)	80.1	(8.4)	77.5	(8.0)	81.5	(8.2)
Age group, *n* (%)						
65–74 years	8403	(28%)	5787	(40%)	4499	(22%)
75–84 years	11,519	(39%)	5612	(39%)	7965	(39%)
≥ 85 years	9697	(33%)	3051	(21%)	7850	(39%)
Sex, *n* (%)						
Male	17,687	(60%)	8853	(61%)	12,314	(61%)
Median treatment period, days (IQR)	–	–	7	(4–9)	14	(9–25)
Death, *n* (%)	–	–	–	–	2389	(12%)
Comorbidities, *n* (%)						
Diabetes mellitus	3134	(11%)	1950	(13%)	2032	(10%)
Chronic obstructive pulmonary disease	3527	(12%)	2426	(17%)	2509	(12%)
Dementia	194	(1%)	103	(1%)	135	(1%)
Dialysis	525	(2%)	293	(2%)	382	(2%)
Liver function failure	4506	(15%)	3020	(21%)	2773	(14%)
Rheumatism	845	(3%)	620	(4%)	489	(2%)
Cancer	2718	(9%)	2284	(16%)	1322	(7%)
Prescriptions during previous 6 months, *n* (%)						
Oral antibiotics	2682	(9%)	2244	(16%)	1586	(8%)
Inhaled steroids	1691	(6%)	1279	(9%)	1155	(6%)
Angiotensin-converting enzyme inhibitors	882	(3%)	505	(3%)	586	(3%)
Statins	2634	(9%)	1678	(12%)	1583	(8%)

*CAP* community-acquired pneumonia, *SD* standard deviation, *IQR* interquartile range

### All-cause CAP treatment costs

**Fig. 2:**
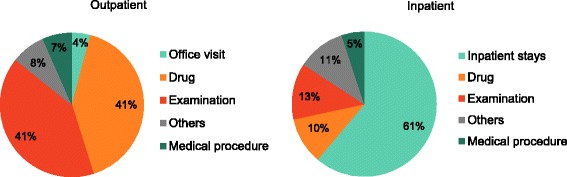
The specific treatment costs as percentages of the total treatment cost

The median treatment costs were $346 (IQR: $195–551) per outpatient episode and $4851 (IQR: $3313–7669) per inpatient episode. Drug costs and laboratory test costs accounted for 82% of the outpatient CAP episode costs, while 61% of inpatient treatment costs were related to the inpatient stays (Fig. 2). Approximately 58% of the episodes (20,145/34,764) involved hospitalization in the general ward, and relatively few episodes involved ICU treatment (0.5%, 169/34,764). However, the treatment costs of ICU episodes were approximately 2.6× higher, compared to general ward episodes ($12,728 [IQR: $8059–21,512] vs. $4824 [IQR: $3301–7591], respectively). The combined total cost of CAP during the study period was $136,575,963, with outpatient episodes accounting for 6% of these costs, general ward episodes accounting for 92%, and ICU episodes accounting for 2%. The median treatment costs for cases of in-hospital mortality (*n* = 2389) were significantly higher than cases with survival until discharge ($6474 [IQR: $3372–10,639] vs. $4741 [IQR: $3308–7287], respectively).

**Fig. 3:**
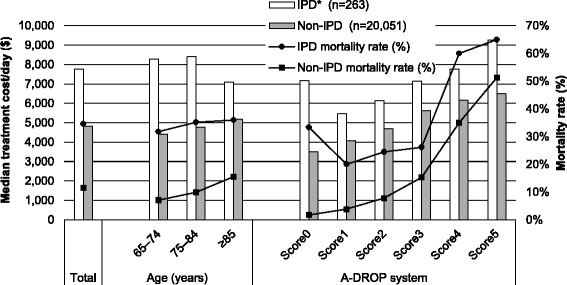
The median all-cause treatment costs of community-acquired pneumonia episodes with or without invasive pneumococcal disease episodes. *IPD: invasive pneumococcal disease

CAP severity data were available for 83% of the inpatient episodes (16,931/20,314). The mean treatment costs of CAP episodes according to severity score are shown in Table 2. Higher severity was associated with increased treatment costs at the treating hospital, as well as with a higher mortality rate and prolonged treatment period. Approximately 1.3% of inpatient episodes involved IPD (263/20,314), and 4.6% (12/263) of the IPD episodes were treated in the ICU. The mean age of patients with IPD episodes was 80.7 ± 7.5 years, and 67% of the patients were men. The LOS for IPD episodes was longer than the LOS for non-IPD episodes (21 days vs. 14 days). The mortality rate for non-IPD episodes was 11%, compared to 35% for IPD episodes. Additional detailed data are shown in Additional file 1: Table S4. The median treatment cost per IPD episode was $7766 (IQR: $4411–12,762), and that cost was higher than the cost for non-IPD episodes ($4831 [IQR: $3304–7603]). Approximately 62% of the IPD episodes (162/263) had available CAP severity data. Cases with a severity score of 5 had the highest IPD rate (8%). Figure 3 shows the median treatment costs and mortality rates for IPD and non-IPD episodes. IPD episodes had higher median treatment costs and an increased mortality rate. Compared to non-IPD episodes, the mortality rate for IPD episodes was increased by 11–32%.

**Table 2:** All-cause total treatment costs according to severity and community-acquired pneumonia treatment setting

All cases (*n* = 16,931)	*n*	%	Median cost	Median treatment period (days)	Mortality rate (%)
Score 0	1006	6%	3508	10	2%
Score 1	5514	33%	4085	12	4%
Score 2	5644	33%	4706	14	8%
Score 3	3387	20%	5630	16	15%
Score 4	1126	7%	6175	17	35%
Score 5	254	2%	6578	19	52%
General ward (*n* = 16,809)	*n*	%	Median cost	Median treatment period (days)	Mortality rate (%)
Score 0	1005	6%	3507	10	2%
Score 1	5502	33%	4079	12	4%
Score 2	5602	33%	4679	13	8%
Score 3	3353	20%	5597	16	15%
Score 4	1104	7%	6099	17	35%
Score 5	243	1%	6410	18	52%
Intensive care unit (*n* = 122)	*n*	%	Median cost	Median treatment period (days)	Mortality rate (%)
Score 0	1	1%	4556	10	0%
Score 1	12	10%	13,264	34	0%
Score 2	42	34%	11,406	21	24%
Score 3	34	28%	11,763	26	18%
Score 4	22	18%	10,583	18	55%
Score 5	11	9%	14,117	22	55%

### All-cause CAP treatment costs according to risk factors

Total costs were associated with male sex, diabetes, COPD, and liver function failure (Table 3). Approximately 90% of the total costs were spent on general ward episodes for each risk factor. Increasing age was associated with higher hospitalization rates and total treatment costs. CAP episodes that involved dementia and dialysis were also associated with high admission rates. Table 4 shows the median treatment costs per CAP episode, the median treatment periods, and the mortality rates according to the risk factors. Inpatient CAP episodes that involved dementia, dialysis, and rheumatism were associated with high treatment costs (likely because of the prolonged LOS) and high mortality rates. Inpatient CAP episodes that involved cancer were associated with a high mortality rate, a short LOS, and low treatment costs.

**Table 3:** A breakdown of all-cause total treatment costs according to risk factors

	Total episodes	Proportion of CAP episodes (%)			Total costs		Proportion of total costs (%)		
Risk factors	*n*	Outpatient episodes	GW episodes	ICU episodes	$	%	Outpatient episodes	GW episodes	ICU episodes
Total	34,764	41.6%	57.9%	0.5%	136,575,963	100.0%	5.6%	92.2%	2.2%
Sex									
Male	21,167	41.8%	57.6%	0.6%	83,776,408	61.3%	5.8%	91.7%	2.5%
Female	13,597	41.2%	58.5%	0.4%	52,799,556	38.7%	5.4%	93.0%	1.6%
Age group									
65–74 years	10,286	56.3%	43.3%	0.5%	31,437,785	23.0%	10.8%	85.5%	3.7%
75–84 years	13,577	41.3%	58.1%	0.6%	54,022,674	39.6%	5.6%	92.4%	2.0%
≥ 85 years	10,901	28.0%	71.6%	0.4%	51,115,505	37.4%	2.5%	96.1%	1.3%
Comorbidities									
Diabetes mellitus	3982	49.0%	50.3%	0.8%	14,534,072	10.6%	8.9%	88.2%	3.0%
COPD	4935	49.2%	50.3%	0.6%	16,749,527	12.3%	8.2%	89.6%	2.3%
Liver function failure	5793	52.1%	47.4%	0.5%	19,154,382	14.0%	9.2%	88.5%	2.2%
Dementia	238	43.3%	55.9%	0.8%	936,780	0.7%	5.5%	90.0%	4.5%
Dialysis	675	43.4%	55.6%	1.0%	3,592,463	2.6%	9.1%	83.9%	7.0%
Rheumatism	1109	55.9%	43.7%	0.4%	3,533,324	2.6%	11.4%	84.9%	3.7%
Cancer	3606	63.3%	36.4%	0.3%	9,999,506	7.3%	18.6%	80.0%	1.4%
Prescriptions during previous 6 months									
Oral antibiotics	3830	58.6%	40.8%	0.6%	11,646,586	8.5%	13.7%	82.8%	3.5%
Inhaled steroids	2434	52.5%	46.6%	0.8%	7,329,868	5.4%	9.7%	86.9%	3.4%
ACE-I	1091	46.3%	53.0%	0.7%	4,087,905	3.0%	7.0%	90.8%	2.2%
Statins	3261	51.5%	47.7%	0.9%	10,744,840	7.9%	9.0%	87.2%	3.9%

*CAP* community-acquired pneumonia, *GW* general ward, *ICU* intensive care unit, *COPD* chronic obstructive pulmonary disease, *ACE-I* angiotensin-converting enzyme inhibitors

**Table 4:** The median treatment costs, treatment periods, and mortality rates according to risk factors

	Median treatment cost per episode ($)			Treatment period (days)			Mortality rate (%)	
Risk factors	Outpatient episodes	GW episodes	ICU episodes	Outpatient episodes	GW episodes	ICU episodes	GW episodes	ICU episodes
Total	346	4824	12,728	7	14	24	12%	30%
Sex								
Male	356	4791	12,518	7	14	22	12%	30%
Female	330	4872	12,792	7	15	27	11%	31%
Age group								
65–74 years	353	4391	19,485	8	12	34	7%	20%
75–84 years	352	4778	11,098	7	14	17	10%	32%
≥ 85 years	323	5162	13,105	6	16	28	16%	39%
Comorbidities								
Diabetes mellitus	384	4878	10,035	8	15	21	10%	30%
COPD	355	4836	11,238	8	14	18	10%	39%
Liver function failure	338	4738	10,029	8	15	21	12%	37%
Dementia	320	5567	21,078	7	17	60	19%	0%
Dialysis	732	6389	23,136	6	15	37	15%	14%
Rheumatism	412	4910	20,673	8	15	34	10%	50%
Cancer	377	4528	8064	8	14	14	15%	50%
Prescriptions during previous 6 months								
Oral antibiotics	375	4821	11,218	8	14	20	13%	46%
Inhaled steroids	346	4622	11,388	7	13	22	8%	30%
ACE-I	298	5124	8778	7	15	19	11%	13%
Statins	352	4705	11,372	7	14	20	7%	21%

*GW* general ward, *ICU* intensive care unit, *COPD* chronic obstructive pulmonary disease, *ACE-I* angiotensin-converting enzyme inhibitors

## Discussion

The present study evaluated the economic burden of CAP in Japan according to severity, as well as the relationship between treatment costs and risk factors. The study found that CAP episodes with hospitalization were 14× more expensive, compared to outpatient episodes, and that stay-related costs accounted for 61% of inpatient treatment costs. It also found that CAP severity, male sex, diabetes, COPD, and liver function failure were associated with high total treatment costs. Furthermore, CAP episodes that involved dementia, dialysis, and rheumatism were associated with high treatment costs, which was likely because of the high admission rate. However, CAP episodes that involved cancer were associated with low inpatient treatment costs, which was likely related to the high mortality rate and short LOS.

Although it was observed that severe CAP, based on the A-DROP system, was associated with high treatment costs, no significant relationship was observed between cost and CURB-65 in a prospective non-interventional clinical study [34]. Another study revealed that total CAP treatment costs among inpatients increased for PSI classes I–III and reached a plateau at classes IV–V [23]. Thus, the relationship between CAP severity and treatment costs may differ depending on the severity score that is used.

The most important factor that influenced treatment cost was hospitalization, which accounted for 94% of total costs. Furthermore, treatment of episodes in the general ward was associated with high total treatment costs, while treatment of episodes in the ICU only accounted for 2% of total costs, despite these episodes clearly involving serious cases (i.e. high treatment costs, high mortality rate, and prolonged LOS). Interestingly, the costs of drug treatment and laboratory testing did not account for a large proportion of the total inpatient costs, and a prospective observational study in a Spanish public tertiary hospital also confirmed that the costs could be broken down into room costs (69%), drug costs (13%), laboratory costs (12%), and diagnostic procedures (6%) [15]. Therefore, the economic burden of CAP could be reduced by decreasing the number of admissions.

A previous study revealed that 61% of mild CAP cases were admitted to a hospital [34], although the rate was only 6% in the present study. This is likely related to differences in the definitions that are used for the CURB-65 and A-DROP systems, as the A-DROP system has one mild category (score 0) and the CURB-65 system has two mild categories (scores 0 and 1). When the CURB-65 system was applied, the results indicated that 34% of mild CAP cases were hospitalized, which suggests that the A-DROP system may underestimate the proportion of mild CAP cases. Thus, the present study’s results highlight the importance of minimizing the hospitalization of mild CAP cases, and other previous studies have also provided similar findings [14, 35–37].

In the present study, the cost per inpatient episode tended to increase with age, although a Dutch administrative database study revealed that the median treatment cost per episode in the general ward remained relatively stable at approximately $5600 for patients who were ≥50 years old [21]. This difference is likely related to the fact that the LOS increased with age in the present study, but was stable in the Dutch study. For Dutch outpatient episodes, the cost was approximately $850 per episode for patients who were 50–84 years old, while the costs were approximately 50% lower for patients who were ≥85 years old. However, in the present study, the treatment cost per CAP episode only decreased slightly for patients who were ≥85 years old. One possible explanation for this difference is that Dutch patients who were ≥85 years old had shorter outpatient treatments, as most patients in this age group were hospitalized.

Based on the findings of the present study, prophylactic treatment (e.g. oral hygiene or pneumococcal vaccination) is recommended to reduce treatment costs for patients who are male and/or have comorbidities (e.g. diabetes, COPD, and liver function failure). In addition, patients with dementia, dialysis, or rheumatism may be an important group to target, given their high treatment costs per CAP episode.

The present study determined the exact CAP treatment costs using a nationally representative data set, while previous studies were limited by only evaluating patients at a few hospitals. In addition, the present study’s calculations were not influenced by changing costs based on the patient’s income or insurance status, as Japanese medical service fees are determined based on a payment list that is approved by the government. Furthermore, healthcare claims data that are used for reimbursement can provide an accurate record of the provided medical services.

The study calculated the all-cause total healthcare costs according to CAP severity and risk factors. This is because the treatment of underlying disease could not be separated from the treatment of CAP exacerbation. By comparing patients with or without CAP, further study is planning to estimate the additional costs of CAP. The treatment costs of CAP episodes that were encountered by general practitioners were not estimated, as the study’s database only included hospital records. However, the authors speculate that outpatient treatment costs of CAP episodes are comparable between hospitals and clinics. This is because almost all residents of Japan are covered by the national health insurance system, which provides equal reimbursement for hospital-based outpatient visits and clinic visits. The reimbursement rates are pre-determined by the Japanese government, and even patients who visit a large hospital without a referral would only make an extra out-of-pocket payment of < $72. However, the study’s database mainly included small- or middle-sized hospitals, which suggests that patients would incur similar treatment costs at a hospital or clinic if they received the same treatment. Moreover, patients can select any hospital or clinic throughout Japan, regardless of the severity of their disease. Therefore, it is expected that there would be little difference in the outpatient treatment costs of CAP episodes at hospitals and clinics.

The present study also has several limitations. First, CAP cases that were caused by *Streptococcus pneumoniae* were not able to be identified, as culture results were not available in the database. Therefore, it was difficult to distinguish between specified or unspecified pneumonia. As there is very little evidence regarding IPD-related treatment costs, further studies are needed to more precisely estimate the IPD treatment costs using culture results. Second, compared to outpatients in the database, the hospitalized patients included fewer immunocompromised patients (e.g. patients with cancer and rheumatism). It is possible that many patients with cancer and a poor general status were included in the database. Another explanation is that all elderly people (> 65 years old) can receive the PPV23 vaccine in Japan, although the vaccination rate in 2014 was only 20% (based on vaccine shipment records), which could indicate that high-risk patients had been vaccinated and were less likely to develop CAP or other serious conditions. Third, a claims database was used for the present study, and this type of study is limited by various issues. For example, there can be differences between diagnostic and reimbursement records, and it would be useful to combine data regarding diagnoses and specific medications. However, there is no specific medication for treating liver function failure, and the present study arbitrarily defined these cases as patients who had ≥2 diagnoses before their index date. Therefore, these diagnoses may have been overestimated.

## Conclusion

This study found that severe CAP cases had high rates of mortality and prolonged hospitalization, which were associated with high treatment costs. In addition, many hospitalizations that involved cases of mild CAP were detected. Therefore, to reduce the economic burden of CAP, clinicians should aim to decrease the number of hospitalized mild CAP cases and to prevent the development of severe CAP. For example, prophylactic treatment (e.g. oral hygiene or vaccination) may be effective for elderly patients who are male, ≥ 85 years old, or have comorbidities (e.g. diabetes, COPD, liver function failure, rheumatism, dementia, or dialysis). These findings can be used to identify population(s) that should be targeted by the vaccine program, based on their burden of diseases, and will help to estimate the value of the vaccine program. Furthermore, vaccination coverage remains low in Japan, and efforts should be made to increase pneumococcus vaccination coverage.

## Additional file

Additional file 1: Table S1. Definition of the six categories for the breakdown of total treatment costs. **Table S2.** Definition of comorbidities and their medications. **Table S3.** Definition of drug use. **Table S4.** The median treatment costs and treatment period, and death rate of invasive pneumococcal disease (IPD). (PDF 586 kb)

## Abbreviations

CAP: Community-acquired pneumonia
COPD: Chronic obstructive pulmonary disease
DPC/PDPS: Diagnosis Procedure Combination/Per-Diem Payment System
ICD-10: International Classification of Diseases, 10th revision
ICU: Intensive care unit
IPD: Invasive pneumococcal disease
IQR: Interquartile range
LOS: Length of stay
PPV23: 23-Valent pneumococcal polysaccharide vaccine
PSI: Pneumonia severity index
SD: Standard deviation
