# Supplementary material for: Economic burden of community-acquired pneumonia among elderly patients: a Japanese perspective
Source: Pneumonia (Nathan). 2017 Dec 5;9:19. doi: 10.1186/s41479-017-0042-1 (PMC5715537; doi:10.1186/s41479-017-0042-1)
Supplement: Additional file 1: Table S1. — Definition of the six categories for the breakdown of total treatment costs. Table S2. Definition of comorbidities and their medications. Table S3. Definition of drug use. Table S4. The median treatment costs and treatment period, and death rate of invasive pneumococcal disease (IPD). (PDF 586 kb) [file 41479_2017_42_MOESM1_ESM.pdf]

Additional file 1

Table S1: Definition of the six categories for the breakdown of total treatment costs

Category	Medical fee name	Medical fee code
Hospital charge and office visit	First visit and revisit fee	A0
	Hospital charge	A1 - 4
Drug	Drug fee	C2, D5, E3,F2, G1, H1, I1, J3, K94, L2
Examination	Examination	D0 - 4
	Imaging	E0 - 2
	Pathological diagnosis	N
Medical procedure	Medical management	B
	Prescribing fee	F0, 1, 4, 5
	Injection	G0
	Procedure	J0 - 2
Others	In-home medical care	C0, 1
	Medical material	C3, D6, E4, F3, G2, J4, K95, L3
	Rehabilitation	H0
	Speciaalized psychiatric therapy	I0
	Surgery	K0 -93
	Anesthesia	L0, 1
	Radiation therapy	M

Table S2: Definition of comorbidities and their medications

Disease name (ICD-10)	Medication	ATC code
Diabetes mellitus (E11-14)	Insulin lispro	A10AB04
		A10AC04
		A10AD04
	Insulin aspart	A10AB05
		A10AD05
	Insulin glulisine	A10AB06
	Insulin glargine	A10AE04
	Insulin detemir	A10AE05
	Insulin degludec (genetical recombination) - insulin aspart (genetical recombination) mixt	A10AD06
	Insulin degludec	A10AE06
	Liraglutide	A10BJ02
	Exenatide	A10BJ01
	Lixisenatide	A10BJ03
	Tolbutamide	A10BB03
	Chlorpropamide	A10BB02
	Acetohexamide	A10BB31
	Glyclopyramide	-
	Glibenclamide	A10BB01
	Gliclazide	A10BB09
	Glimepiride	A10BB12
	Buformin hydrochloride	A10BA03
	Metformin hydrochloride	A10BA02
	Pioglitazone hydrochloride	A10BG03
	Acarbose	A10BF01
	Voglibose	A10BF03
	Miglitol	A10BF02
	Nateglinide	A10BX03
	Mitiglinide calcium hydrate	A10BX08
	Repaglinide	A10BX02
	Sitagliptin phosphate	A10BH01
	Vildagliptin	A10BH02
	Alogliptin benzoate	A10BH04
	Linagliptin	A10BH05
	Teneligliptin hydrobromide hydrate	-
	Anagliptin	-
	Saxagliptin hydrate	A10BH03
	Pioglitazone - metformin mixt	A10BD05
	Pioglitazone - glimepiride mixt	A10BD06
	Mitiglinide calcium hydrate - voglibose mixt	-
	Alogliptin benzoate - pioglitazone hydrochloride mixt	A10BD09
	Ipragliflozin L-proline	-
	Tofogliflozin	-
	Dapagliflozin propanediol	A10BK01
	Luseogliflozin hydrate	-
	Canagliflozin	A10BK02
Chronic obstructive pulmonary disease (J42-44)	Ipratropium bromide	R03BB01
	Oxitropium bromide	R03BB02
	Tiotropium bromide hydrate	R03BB04
	Olodaterol hydrochloride	R03AC19
	Glycopyrrolate	R03BB06
	Glycopyrronium bromide - indacaterol maleate mixt	R03AL04

	Salbutamol sulfate	R03AC02
	Terbutaline sulfate	R03AC03
	Procaterol hydrochloride hydrate	R03AC16
	Tulobuterol hydrochloride	R03AC11
	Fenoterol hydrobromide	R03AC04
	Clenbuterol hydrochloride	R03AC14
	Salmeterol xinafoate	R03AC12
	Salmeterol xinafoate - fluticasone propionate mixt	R03AK06
	Formoterol fumarate hydrate	R03AC13
	Budesonide - formoterol fumarate dihydrate mixt	R03AK07
	Fluticasone propionate - formoterol fumarate hydrate mixt	R03AK11
	Indacaterol maleate	R03AC18
	Glycopyrronium bromide - indacaterol maleate mixt	R03AL04
	Aminophylline hydrate	R03DA05
	Theophylline	R03DA04
	Fluticasone propionate	R03BA05
	Fluticasone furoate	R03BA09
	Salmeterol xinafoate - fluticasone propionate mixt	R03AK06
	Fluticasone propionate - formoterol fumarate hydrate mixt	R03AK11
	Fluticasone furoate - vilanterol trifenatate mixt	R03AK10
	Budesonide	R03BA02
	Budesonide - formoterol fumarate dihydrate mixt	R03AK07
	Ciclesonide	R03BA08
	Mometasone furoate hydrate	R03BA07
	Mometasone furoate	R03BA07
Dementia (F00)	Donepezil hydrochloride	N06DA02
	Memantine hydrochloride	N06DX01
	Galantamine hydrobromide	N06DA04
	Rivastigmine	N06DA03
Rheumatism (M059, M060, M068, M069)	Penicillamine	M01CC01
	Lobenzarit sodium	-
	Actarit	-
	Bucillamine	M01CC02
	Sulfasalazine	A07EC01
	Methotrexate	L04AX03
	Leflunomide	L04AA13
	Iguratimod	-
	Tacrolimus	L04AD02
	Mizoribine	-
	Tofacitinib citrate	L04AA29
	Etanercept	L04AB01
	Infliximab	L04AB02
	Tocilizumab	L04AC07
	Adalimumab	L04AB04
	Abatacept	L04AA24
	Golimumab	L04AB06
	Certolizumab pegol	L04AB05
	Gold sodium thiomalate	M01CB01
	Auranofin	M01CB03
	Prednisolone farnesylate	H02AB06
	Azathioprine	L04AX01
	Methylprednisolone acetate	H02AB04
	Methylprednisolone sodium succinate	H02AB04

	Methylprednisolone	H02AB04
	Betamethasone acetate - betamethasone sodium phosphate mixt	-
	Betamethasone sodium phosphate	H02AB01
	Betamethasone	H02AB01
	Prednisolone sodium succinate	H02AB06
	Prednisolone	H02AB06
	Hydrocortisone sodium succinate	H02AB09
	Hydrocortisone	H02AB09
	Triamcinolone acetonide	H02AB08
	Triamcinolone	H02AB08
	Dexamethasone sodium phosphate	H02AB02
	Dexamethasone metasulfobenzoate sodium	H02AB02
	Dexamethasone palmitate	H02AB02
	Dexamethasone	H02AB02
	Cortisone acetate	H02AB10
Cancer (C00-99, D00-09)	Cyclophosphamide	L01AA01
	Busulfan	L01AB01
	Melphalan	L01AA03
	Estramustine phosphate sodium hydrate	L01XX11
	Temozolomide	L01AX03
	Mercaptopurine	L01BB02
	Methotrexate	L01BA01
	Tegafur	L01BC03
	Fluorouracil	L01BC02
	Doxifluridine	-
	Capecitabine	L01BC06
	Cytarabine ocfosphate hydrate	L01BC01
	Hydroxyurea	L01XX05
	Fludarabine phosphate	L01BB05
	Tegafur - uracil mixt	L01BC53
	Tegafur - gimeracil - oteracil potassium mixt	L01BC53
	Etoposide	L01CB01
	Procarbazine hydrochloride	L01XB01
	Tamoxifen citrate	L02BA01
	Sobuzoxane	-
	Flutamide	L02BB01
	Tretinoin	L01XX14
	Toremifene citrate	L02BA02
	Bicalutamide	L02BB03
	Anastrozole	L02BG03
	Imatinib mesylate	L01XE01
	Exemestane	L02BG06
	Gefitinib	L01XE02
	Tamibarotene	-
	Letrozole	L02BG04
	Erlotinib hydrochloride	L01XE03
	Sorafenib tosylate	L01XE05
	Sunitinib malate	L01XE04
	Thalidomide	L04AX02
	Dasatinib	L01XE06
	Nilotinib hydrochloride hydrate	L01XE08
	Lapatinib ditosylate	L01XE07
	Everolimus	L01XE10
	Lenalidomide hydrate	L04AX04
	Vorinostat	L01XX38

Crizotinib	L01XE16
Axitinib	L01XE17
Pazopanib hydrochloride	L01XE11
Regorafenib hydrate	L01XE21
Afatinib dimaleate	L01XE13
Enzalutamide	L02BB04
Alectinib hydrochloride	L01XE36
Abiraterone acetate	L02BX03
Ruxolitinib phosphate	L01XE18
Sirolimus	L04AA10
Bosutinib hydrate	L01XE14
Vemurafenib	L01XE15
Pomalidomide	L04AX06
Lenvatinib mesylate	L01XE29
Panobinostat lactate	L01XX42
Vandetanib	L01XE12
Coriolus versicolor polysaccharide	-
Ubenimex	-
Anagrelide hydrochloride hydrate	L01XX35
Trifluridine - tipiracil hydrochloride mixt	L01BC59
Carmustine	L01AD01
Tegafur	L01BC03
Fluorouracil	L01BC02
Bleomycin sulfate	L01DC01
Cyclophosphamide	L01AA01
Ifosfamide	L01AA06
Busulfan	L01AB01
Nimustine hydrochloride	L01AD06
Dacarbazine	L01AX04
Ranimustine	L01AD07
Melphalan	L01AA03
Temozolomide	L01AX03
Bendamustine hydrochloride	L01AA09
Streptozocin	L01AD04
Enocitabine	-
Gemcitabine hydrochloride	L01BC05
Cytarabine	L01BC01
Fludarabine phosphate	L01BB05
Pemetrexed sodium hydrate	L01BA04
Nelarabine	L01BB07
Clofarabine	L01BB06
Mitomycin	L01DC03
Dactinomycin	L01DA01
Bleomycin hydrochloride	L01DC01
Peplomycin sulfate	-
Aclarubicin hydrochloride	L01DB04
Daunorubicin hydrochloride	L01DB02
Doxorubicin hydrochloride	L01DB01
Pirarubicin hydrochloride	L01DB08
Epirubicin hydrochloride	L01DB03
Idarubicin hydrochloride	L01DB06
Amrubicin hydrochloride	L01DB10
Gemtuzumab ozogamicin	L01XC05
Vincristine sulfate	L01CA02
Vinblastine sulfate	L01CA01
Vindesine sulfate	L01CA03

Irinotecan hydrochloride	L01XX19
Docetaxel	L01CD02
Paclitaxel	L01CD01
Vinorelbine tartrate	L01CA04
Nogitecan hydrochloride	L01XX17
Cabazitaxel acetonate	L01CD04
Asparaginase	L01XX02
Cisplatin	L01XA01
Mitoxantrone hydrochloride	L01DB07
Carboplatin	L01XA02
Pentostatin	L01XX08
Nedaplatin	-
Trastuzumab (genetical recombination)	L01XC03
Rituximab	L01XC02
Cladribine	L01BB04
Arsenic trioxide	L01XX27
Oxaliplatin	L01XA03
Bortezomib	L01XX32
Bevacizumab	L01XX32
Ibritumomab tiuxetan	V10XX02
Cetuximab	L01XC06
Miriplatin hydrate	-
Panitumumab	L01XC08
Temsirolimus	L01XE09
Azacitidine	L01BC07
Eribulin mesylate	L01XX41
Fulvestrant	L02BA03
Mogamulizumab	L01XC25
Ofatumumab	L01XC10
Pertuzumab	L01XC13
Trastuzumab emtansine	L01XC14
Nivolumab	L01XC17
Alemtuzumab	L04AA34
Ramucirumab	L01XC21
Ipilimumab	L01XC11
Trabectedin	L01CX01
Picibanil	-
Lentinan	L03AX01
Porfimer sodium	L01XD01
Talaporfin sodium	-
Talc	-

Table S3: Definition of drug use

Therapeutic category	Generic name	ATC code
Oral antibiotics	Cortisone	H02AB10
	Hydrocortisone	H02AB09
	Fludrocortisone	H02AA02
	Prednisolone	H02AB06
	Methylprednisolone	H02AB04
	Triamcinolone	H02AB08
	Dexamethasone	H02AB02
	Betamethasone	H02AB01
Inhaled steroids	Beclometasone	R01AD01
	Fluticasone	R03BA05
	Salmeterol and fluticasone	R03AK06
	Formoterol and fluticasone	R03AK11
	Vilanterol and fluticasone furoate	R03AK10
	Budesonide	R03BA02
	Formoterol and budesonide	R03AK07
	Ciclesonide	R03BA08
	Mometasone	R03BA07
Angiotensin-converting enzyme inhibitors	Captopril	C09AA01
	Enalapril	C09AA02
	Alacepril	-
	Delapril	C09AA12
	Cilazapril	C09AA08
	Lisinopril	C09AA03
	Benazepril	C09AA07
	Imidapril	C09AA16
	Temocapril	C09AA14
	Quinapril	C09AA06
	Trandolapril	C09AA10
	Perindopril	C09AA04
Statins	Pravastatin	C10AA03
	Simvastatin	C10AA01
	Fluvastatin	C10AA04
	Atorvastatin	C10AA05
	Pitavastatin	C10AA08
	Rosuvastatin	C10AA07
	Amlodipine besilate - atorvastatin calcium mixt	C10BX03

Table S4: The median treatment costs and treatment period, and death rate of invasive pneumococcal disease (IPD)

IPD*		n	%	Median	(	25%tile	-	75%tile	)	Median treatment period	(	25%tile	-	75%tile	)	Death (%)
Total		263	-	864,405	(	490,890	-	1,420,456	)	21	(	11	-	40	)	35%
Sex																
	Men	177	67%	846,658	(	484,008	-	1,420,456	)	21	(	10	-	40	)	34%
	Female	86	33%	890,625	(	518,805	-	1,420,037	)	19	(	12	-	39	)	35%
Age																
	65-74	63	24%	919,922	(	478,085	-	1,420,037	)	17	(	10	-	38	)	32%
	75-84	111	42%	934,653	(	525,765	-	1,796,859	)	23	(	11	-	43	)	35%
	85-	89	34%	789,909	(	433,733	-	1,208,130	)	21	(	10	-	37	)	36%
Death		91	35%	1,182,284	(	582,960		1,956,972	)	24	(	9	-	41	)	-
Risk factor																
	Diabetes mellitus	50	19%	953,863	(	525,765	-	1,396,733	)	27	(	13	-	41	)	34%
	Chronic obstructive pulmonary disease	32	12%	1,115,365	(	541,494	-	2,191,297	)	24	(	15	-	48	)	41%
	Dementia	5	2%	846,658	(	740,062	-	1,559,037	)	22	(	12	-	25	)	40%
	Dialysis	10	4%	2,237,032	(	1,300,203	-	2,433,761	)	44	(	27	-	61	)	20%
	Liver dysfunction	59	22%	936,221	(	572,568	-	1,398,894	)	25	(	15	-	42	)	27%
	Rheumatism	10	4%	634,026	(	402,898	-	1,239,441	)	15	(	10	-	33	)	10%

	Cancer	26	10%	724,598	(	420,207	-	1,301,421	)	23	(	10	-	35	)	38%
Prescription																
	Oral steroid	39	15%	919,922	(	573,590	-	1,485,356	)	21	(	12	-	35	)	41%
	Inhaled steroid	12	5%	814,616	(	487,449	-	1,674,051	)	19	(	13	-	26	)	33%
	Angiotensin-converting enzyme inhibitor	15	6%	678,229	(	433,733	-	1,249,058	)	16	(	10	-	67	)	53%
	Statin	27	10%	592,603	(	433,733	-	1,300,203	)	15	(	10	-	27	)	37%
A-DROP system																
	Score0	6	2%	798,517	(	421,621	-	1,376,402	)	21	(	10	-	66	)	33%
	Score1	30	11%	607,963	(	390,312	-	875,273	)	14	(	10	-	21	)	20%
	Score2	45	17%	684,022	(	442,720	-	1,025,908	)	15	(	10	-	31	)	24%
	Score3	46	17%	794,514	(	536,953	-	1,208,130	)	18	(	12	-	33	)	26%
	Score4	15	6%	864,405	(	321,850	-	1,420,456	)	15	(	4	-	35	)	60%
	Score5	20	8%	1,031,698	(	580,013	-	1,906,766	)	24	(	7	-	40	)	65%
Non-IPD		n	%	Median	(	25%tile	-	75%tile	)	Median treatment period	(	25%tile	-	75%tile	)	Death (%)
Total		20,051	-	537,718	(	367,713	-	846,159	)	14	(	9	-	25	)	11%
Sex																
	Men	12,137	61%	534,257	(	364,460	-	844,836	)	14	(	9	-	24	)	12%
	Female	7,914	39%	542,651	(	372,745	-	849,333	)	15	(	10	-	25	)	11%
Age																
	65-74	4,436	22%	489,800	(	342,244	-	763,288	)	12	(	9	-	20	)	7%

	75-84	7,854	39%	532,321	(368,579 - 846,836)	14 (9 - 24)	10%
	85-	7,761	39%	574,988	(383,921 - 887,538)	16 (10 - 28)	16%
Death		2,298	11%	711,031	(371,484 - 1,161,827)	16 (7 - 35)	-
Risk factor							
	Diabetes mellitus	1,982	10%	544,691	(373,956 - 863,259)	15 (10 - 25)	10%
	Chronic obstructive pulmonary disease	2,477	12%	539,722	(376,853 - 806,698)	14 (9 - 22)	10%
	Dementia	130	1%	610,415	(348,911 - 961,158)	18 (11 - 33)	18%
	Dialysis	372	2%	708,298	(443,195 - 1,127,676)	15 (10 - 27)	15%
	Liver dysfunction	2,714	14%	527,049	(363,135 - 827,768)	14 (9 - 24)	12%
	Rheumatism	479	2%	546,507	(381,025 - 875,446)	15 (10 - 27)	11%
	Cancer	1,296	6%	504,013	(337,873 - 823,538)	14 (9 - 23)	15%
Prescription							
	Oral steroid	1,547	8%	536,393	(369,636 - 833,278)	14 (9 - 24)	13%
	Inhaled steroid	1,143	6%	521,174	(363,135 - 769,968)	13 (9 - 21)	8%
	Angiotensin-converting enzyme inhibitor	571	3%	571,941	(386,059 - 899,597)	15 (10 - 26)	9%
	Statin	1,556	8%	526,283	(368,144 - 803,272)	14 (9 - 23)	7%
A-DROP system							
	Score0	1,000	5%	389,196	(297,711 - 541,816)	10 (8 - 15)	2%
	Score1	5,484	27%	454,057	(325,683 - 656,689)	12 (9 - 18)	4%
	Score2	5,599	28%	522,740	(377,431 - 775,406)	13 (9 - 22)	8%
	Score3	3,341	17%	624,617	(434,094 - 958,233)	16 (10 - 27)	15%

Score4	1,111	6%	686,770	(	439,941	-	1,018,905	)	17	(	10	-	29	)	35%
Score5	234	1%	723,190	(	399,341	-	1,208,718	)	18	(	7	-	35	)	51%

*IPD: invasive pneumococcal disease
